# Tunicates as Sources of High-Quality Nutrients and Bioactive Compounds for Food/Feed and Pharmaceutical Applications: A Review

**DOI:** 10.3390/foods12193684

**Published:** 2023-10-07

**Authors:** Pingping Gao, Heng Yen Khong, Wenhui Mao, Xiaoyun Chen, Lingxiang Bao, Xinru Wen, Yan Xu

**Affiliations:** 1 Faculty of Applied Sciences, Universiti Teknologi MARA, Sarawak Branch, Kota Samarahan 94300, Malaysia; 2 School of Food and Pharmacy, Zhejiang Ocean University, Zhoushan 316022, Chinaxuyan@zjou.edu.cn (Y.X.)

**Keywords:** tunicate, chemical composition, nutrients, food, feed, bioactive compounds, pharmaceutical applications

## Abstract

Tunicates are widely distributed worldwide and are recognized as abundant marine bioresources with many potential applications. In this review, state-of-the-art studies on chemical composition analyses of various tunicate species were summarized; these studies confirmed that tunicates contain nutrients similar to fish (such as abundant cellulose, protein, and ω-3 fatty acid (FA)-rich lipids), indicating their practical and feasible uses for food or animal feed exploration. However, the presence of certain toxic elements should be evaluated in terms of safety. Moreover, recent studies on bioactive substances extracted from tunicates (such as toxins, sphingomyelins, and tunichromes) were analyzed, and their biological properties were comprehensively reviewed, including antimicrobial, anticancer, antioxidant, antidiabetic, and anti-inflammatory activities. In addition, some insights and prospects for the future exploration of tunicates are provided which are expected to guide their further application in the food, animal feed, and pharmaceutical industries. This review is critical to providing a new pathway for converting the common pollution issues of hydroponic nutrients into valuable marine bioresources.

## 1. Introduction

In recent years, the world food crisis has become more serious due to natural disasters such as floods and droughts and the outbreak of the novel coronavirus (2019-nCoV) [1]. Researchers have been seeking alternative protein sources in various regions to address this global issue [2]. Marine bioresources, such as fish, seaweed, and shrimp, account for a large part of foods for human beings, and it is critical to tackle the global food crisis by producing cheap and high-quality protein sources. Among these sources, tunicates, representing a new type of marine bioresource containing high contents of good-quality protein, can be a supplemental food source, thus helping alleviate the food crisis [3].

Tunicates create a severe fouling problem in the aquaculture industry, easily settling on solid matter and producing a large volume of biomass living in the ocean. They are highly diverse marine invertebrates, with approximately 3000 recognized species worldwide [4]. Tunicates grow quickly, have a short maturity period, and can quickly occupy artificial facilities such as buoys, bottoms, and cages. With the development of ocean shipping and aquaculture industries, tunicates are more likely to be brought into new environments. Compared with sponges, shellfish, seaweed, and finned fish, they exhibit a faster growth speed and greater proliferation and survival abilities, and have become a significant fouling animal species in the sea. They compete for food and habitat space with economic shellfish, such as mussels, scallops, pearl oysters, and oysters. Furthermore, tunicates prey on their larvae and exclude juveniles, interfere with the secretion of foot filaments, hinder the opening of the shell, and affect normal physiological activities such as feeding and respiration, thus resulting in the slow growth of cultured objects, increased mortality, and decreased yield. With the expansion of the cultured sea area, tunicates may also become carriers of harmful algae diffusion. In addition, the attachment of a tunicate inevitably blocks the mesh of the cage, reducing the exchange of water between the internal and external environments and the dissolved oxygen content in the water body, thus damaging aquaculture equipment and polluting the local water environment [5].

Until now, fouling tunicates in aquaculture have always been removed from infrastructure and discarded in the sea, which both contaminates the water and causes serious eutrophication issues. Recently, more interest has arisen in valorizing waste marine byproducts into valuable products [6]; as a result, these abundant marine bioresources are exploited for material, food, animal feed, and energy production rather than being disposed of in the ocean. For example, most tunicate species are not edible, but some solitary stolidobranchs in the Styelidae and Pyuridae families are harvested wild or cultured for seafood, and the main species include *Halocynthia aurantium*, *Halocynthia roretzi*, *Microcosmus hartmeyeri*, *Microcosmus sabatieri*, *Microcosmus vulgaris*, *Polycarpa pomaria*, *Pyura chilensis*, *Styela clava*, and *Styela plicata*, which may be eaten raw, cooked, dried, or pickled [3]. *Ciona intestinalis* has been developed as a sustainable protein source for the formulation of animal feed [7]. With over 1000 isolated bioactive molecules and numerous reported applications [8] due to antitumoral, antiviral, antifungal, anti-inflammatory, and antibacterial properties [9], tunicates are an up-and-coming source of marine natural products (MNPs) for potential pharmaceutical applications. Some edible tunicate species, such as *Halocynthia roretzi and Halocynthia aurantium*, have been cultivated and consumed as delicious foods in many countries. Exploring the full application of tunicates (mainly the edible species) will contribute to marine biodiversity and increase cultural and economic importance [10]. Moreover, tunicates, as incredibly effective filter-feeding creatures, can be useful bioindicators of environmental pollution in the marine environment, and they can concentrate and accumulate dangerous toxicants, mainly heavy metals (such as iron, vanadium, titanium, cadmium, and chromium), even at low concentrations [11]. The accumulation of heavy metal ions may introduce risky factors when tunicates are consumed as seafood.

Herein, a review was conducted to introduce the life cycle, chemical composition, nutritional profile, bioactive compounds, and biological properties of tunicates, thus guiding the further exploration of their use in the food, animal feed, and pharmaceutical industries. This review will provide a feasible solution to common fouling problems in aquaculture and new insights into converting unexpected marine pollutants to valuable marine bioresources.

## 2. The Tunicates and Its Life Cycle

Tunicates, also known as sea squirts or ascidians, are sessile marine invertebrates that exhibit sac-like structures and engage in filter feeding. They belong to the subphylum Tunicate, classified within the chordate group [12]. Globally, there are approximately 2000 species of marsupials worldwide, with the majority being tunicates. Tunicates are sedentary denizens of the marine benthos, occupying habitats ranging from the intertidal region to a maximum depth of 6000 m [13]. *Styela clava, Styela plicata, Styela canopus, Ciona intestinalis, Molgula manhattensis,* and *Halocynthia roretzi* are common tunicate species. In total, 103 species have existed in China, including 5 in the Bohai Sea, 21 in the Yellow Sea, 24 in the East China Sea, and 53 in the South China Sea. *Halocynthia roretzi* is found along the Sanriku coast on the Pacific side of Japan and in the Sea of Japan north of the Oga Peninsula, as well as on the east and south coasts of Korea. Tunicates exhibit robust growth and proliferate on marine buoys, stones, boat bottoms, floats, and pilings with mild wave action and abundant nutrients. They prefer cold weather and mainly live in cold or temperate regions, with fewer found in tropical regions at smaller sizes.

**Figure 1:**
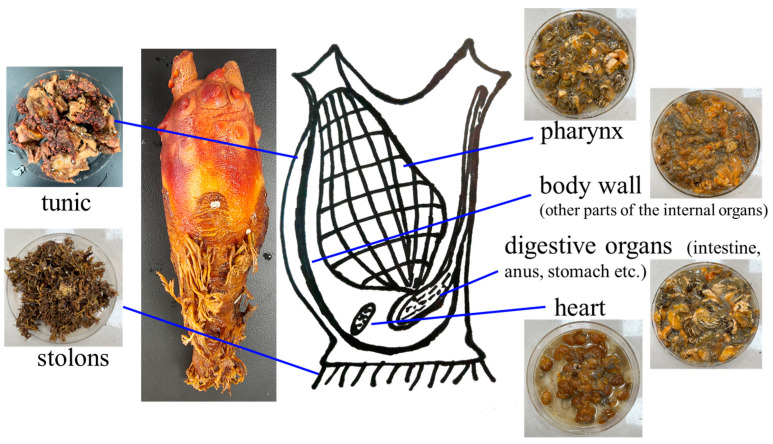
The structure of a tunicate.

These hermaphroditic organisms have a lifespan of two months to a year and show a variety of body hues, including a range of colors from translucent to red, yellow, green, brown, and blue. The structure of the tunicate is shown in Figure 1. As omnivorous filter feeders, when swimming, tunicates use their circular muscle bands to contract and pump water through their oral siphons to filter water for nutrition. Additionally, they have inlet and outlet pipe holes. When water passes through the body, particles ranging in size from 1 µm to 1 mm are trapped in the mucous net and move toward the esophagus. Tunicates use a fine mucous mesh to filter water at a rate more than 1000 times the volume of the organism [14]. They have the highest filtration rate of all marine zooplankton at 15.3 mL/s [15]. Despite the lack of published information about their feeding habits, one study revealed that approximately 80% of their diet is phytoplankton, such as Coccolithophoridae, Silicoflagellata, and Bacillariophyceae, and the rest comprises Radiolaria and Tintinnidae [16].

Tunicates are classified into two types based on their body structure and functional organization, and the majority of tunicates are monoecious. Solitary tunicates live independently of their tunics, while colonial tunicates live physically attached to each other’s tunics [17]. Colonial species brood their young larvae and release mature larvae into the water, while most others spawn eggs. Sperm undergo a period of approximately 24 h in swimming larvae, with an average larval duration of 12–24 h [18]. These species demonstrate swift growth rates, attaining sexual maturity within a few weeks, and possess a stationary adult form that engages in suspension feeding. Their larval stage is transient and nonfeeding [18]. These characteristics result in a high volume of tunicate bioresource in the sea, and finding practical and feasible applications for this new type of marine biomass is critical to both aquaculture and the environment.

## 3. The Chemical Compositions of Tunicates

A chemical composition analysis is a prerequisite for bioresource exploitation, guiding specific applications. Anatomically, all tunicates can be divided into two main parts, i.e., the outer shell and the internal organs. The outer shell is an external supportive tissue that holds the body shape and helps the animals filter sea water; it is commonly called a tunic. In addition, the tunic prohibits predators from attacking. The outer shell contains ~60% cellulose, and nitrogen-containing organic ingredients comprise another ~27% by dry weight [19]. The elemental composition of *Salpa thompsoni* was determined in which moisture was 93.6% (aggregate form) and 92.3% (solitary form). The ash content was as high as 44% of the dry weight. Carbon and nitrogen contents accounted for 17–22% and 3–5% of the dry weight, respectively [20]. Based on the available literature, the chemical compositions of many different tunicate species are listed in Table 1, and different tunicate species and fractions have different chemical compositions [3].

The outer shell and internal organs of the tunicate possess different chemical compositions. Zhao et al. analyzed the chemical compositions of three common tunicate species, *Ciona intestinalis*, *Styela plicata*, and *Ascidia* sp. [21]. The internal organs had higher protein and lipid levels than the outer shells, while the latter were more abundant in cellulose. Of the different species, the internal organs of *Ciona intestinalis* organs contained the highest protein content of 69.32% but the lowest cellulose content of 5.59%. The cellulose content of the outer shell of *Styela plicata* shell was the highest at 57.67%, with the lowest lipid content at 0.35% (Table 2). The quality of the proteins in these tunicate species was evaluated by comparing them with whole chicken egg protein. The ratios of essential amino acids (EAAs) to the total amino acids (TAAs) in all the internal organs were high and similar, reaching 32–50%. The essential amino acid index (EAAI) represents the similarity of the sample’s amino acid profile to that of egg protein, and this value was also commonly high, 58–80%, except for *Ascidia* sp. This result indicates that tunicate protein generally exhibits good quality and is suitable for food/feed applications.

**Table 1:** Chemical compositions of different tunicate species.

Tunicates	Water (%)	Ash (%)	Protein (%)	Carbohydrate (%)	Lipids (%)	Reference
*Cyclosalpa affinis*	97.61	62.64 ± 1.91	6.96 ± 2.92	0.91 ± 0.01	1.03 ± 0.08	[22]
*Cyclosalpa* sp.	95.72 ± 0.31	55.21	9.09	0.84	3.73	[22]
*Iasis zonaria*	96.51 ± 0.57	76.42 ± 4.10	10.16 ± 0.77	2.39 ± 0.27	1.68 ± 0.08	[22]
*Salpa fusiformis*	96.17	68.53 ± 2.01	2.57 ± 0.20	0.63 ± 0.03	1.52 ± 0.78	[22]
*Salpa maxima*	96.98 ± 0.31	60	9.94	1.32	3.81	[22]
*Salp unknown*	96.20 ± 0.29	60.21	14.09 ± 0.23	2.14 ± 0.13	3.13 ± 0.75	[22]
*Thalia democratica*	95.04 ± 0.05	70.07 ± 2.05	9.07 ± 1.21	1.11 ± 0.08	1.44 ± 0.26	[22]
*Thetys vagina*	95.05	67.54 ± 4.03	3.86 ± 1.68	0.99 ± 0.11	0.68 ± 0.06	[22]
*Cnemidocarpa verrucosa*	-	38.8 ± 1.70	6.20 ± 0.50	0.70 ± 0.05	4.90 ± 0.90	[23]
*Salpa fusiformis*	-	66.41 ± 3.85	0.50 ± 0.15	0.06 ± 0.02	0.22 ± 0.18	[24]
*Salpa thompsoni*	96.90	54.90	9.20	1.10	5.50	[25]

All ash, protein, carbohydrate, and lipid contents are expressed on a dry basis, and “±” stands for “standard deviation”.

The chemical contents of the two most common edible tunicate species, *Halocynthia roretzi,* and *Halocynthia aurantium*, were previously analyzed [26]. *Halocynthia roretzi* contains 77.5% moisture, 11.3% crude protein, 1.1% crude lipids, 2.5% ash, and 6.6% glycogen. A total of 83% of the extracted lipids are neutral (NL), while 17% are phospholipids (PL). Asp, Glu, and Lys are the major amino acids. The major fatty acids are 14:0, 16:0, 16:1ω7, 18:1ω7, 18:4ω3, 20:5ω3, and 22:6ω3, and the content of ω-3 polyunsaturated fatty acids is 39%. The inorganic ingredients are mainly Na^+^, K^+^, Cl^−^, and PO_4_^3−^. Zhao and his coresearchers determined the principal chemical composition of *Halocynthia roretzi* [27]. *Halocynthia roretzi* has a body size of 12.7 ± 4.5 cm (length) × 6.2 ± 2.6 cm (width) and an average body weight of 70.4 ± 16.7 g. *Halocynthia roretzi* contains 11.55% ash, 38.08% protein, 0.28% lipids, and 46.52% carbohydrates. The chemical composition data obtained in previous studies suggested that the tunicate can be a valuable nutrient source, and its nutritional profile is comparable to certain seafood products, making it suitable for food/feed applications.

## 4. Chemical Constituents of Tunicates

Tunicates are the only animals which produce cellulose and are rich in pure cellulose. For example, high-quality cellulose with a high level of purity, excellent thermal stability, and good mechanical properties has been prepared from various tunicate species, such as *Ciona intestinalis*, *Styela plicata*, *Halocynthia roretzi*, and *Ascidia* sp. [21]. In addition, tunicate celluloses can be further processed into nanocellulose, which can be used to develop many cellulose-based materials, such as food packaging materials, luminescent fibers, strong composites, and renewable sensors, showing great application potential in food and material fields [21,28]. Furthermore, the polysaccharides present in the outer shells of tunicates, mainly cellulose, can be degraded into glucose, which can be further fermented to prepare bioethanol for the energy field.

The internal organs are covered by a shell, including pharyngeal slits, endostyles, gonads, heart, stomach, intestine, and anus. All these organs play essential roles in realizing the full functions of tunicates, such as feeding sea water, digesting organic components, and maintaining other life activities [29]. The internal organs are rich in essential amino acids, minerals, fatty acids, etc., with the essential amino acid content reaching over 90%. The edible parts of *Halocynthia roretzi* contain 14 trace elements such as As, Pb, Cd, Se, Cr, Sn, Li, Ni, Fe, Mn, Cu, Zn, Ca, and Sr. The content of the mineral Zn is much higher than in other marine animals, and the contents of heavy metals are far lower than the national standards for toxic and harmful substances in aquatic products, making it safe to consume. The phospholipid, eicosapentaenoic acid (EPA, 20:5ω3), and docosahexaenoic acid (DHA, 22:6ω3) contents in tunicates are also high. Troedsson et al. reported that [30] abundant good proteins are indicated by high contents of essential and delicious amino acids and healthy marine-derived lipids containing good-quality ω-3 fatty acids. These lipids can serve as a potential fish oil substitute and have been applied to produce biodiesel after esterification [30]. Many studies have mainly focused on extracting and characterizing lipids from various tunicate species. Zhao et al. analyzed the fatty acid composition of three tunicate species, demonstrating that all tunicate internal organs generally have high polyunsaturated fatty acids (PUFA) contents (Table 3) [27]. Culkin and Morris also determined the fatty acid contents of two tunicate species, *Pyrosoma* and *Salpa cylindrica*. These two tunicate species are rich in myristic acid, with 13.9% and 12.6%, respectively. However, the commonly found polyunsaturated acid C22:6 and the polyunsaturated C16 fatty acids in phytoplankton are not abundant [16].

*Halocynthia roretzi* has a total lipid content of 2.0%, 36.6% of which are neutral lipids, 46.2% are phospholipids, and the rest are glycolipids at a concentration of 17.2%. In neutral lipids, triglycerides and free sterols account for 49.0% and 25.8%, respectively. The others include 9.4% diglycerides, 6% monoglycerides, 4.6% free fatty acids, and 5.2% esterified sterols and hydrocarbons. Phosphatidylcholine and phosphatidylethanolamine are the major phospholipids, with concentrations of 48.6% and 32.4%, respectively. The other phospholipids include 9.8% phosphatidylinositol, 5.7% phosphatidylserine, and 3.5% unknown compounds. Eicosapentaenoic acid (21.3%), docosahexaenoic acid (16.3%), palmitic acid (13.8%), and oleic acid (8.5%) are the dominant fatty acids present in the total lipids [31].

Despite the well-established knowledge regarding the presence of biologically active compounds in tunicates, a specific chemical investigation of *Halocynthia aurantium,* an edible marine invertebrate found in the East Sea and commonly consumed in East Asian countries such as Korea and Japan, is still lacking. In a recent study, Chaiwat and his research team analyzed the fatty acid compositions of the tunic of *Halocynthia aurantium*. The results revealed that the tunic primarily consisted of palmitic acids (21.73 ± 2.16%), oleic acid (6.78 ± 0.28%), stearic acid (33.13 ± 3.22%), lauric acid (2.72 ± 0.23%), dihomo α-linolenic acid (4.09 ± 0.36%), DHA (3.38 ± 0.34%), and EPA (3.88 ± 0.31%). Notably, both the outer membrane (OM) and inner membrane (IM) exhibited higher levels of ω-3 PUFAs than ω-6 PUFAs. Furthermore, the levels of EPA were higher than those of DHA [32]. An additional study proved that the fractionated lipids derived from the tunic of *Halocynthia aurantium* consist of significant proportions of various types of lipids, including neutral lipids, glycolipids, and phospholipids. These lipids are primarily composed of saturated fatty acids (SFAs) such as 16:0 and 18:0, monounsaturated fatty acids (MUFAs) such as 16:1ω7 and 18:1ω9, and PUFAs such as 18:2ω6 and 20:5ω3 [33].

*Botryllus schlossen* contains eighteen sterols, the majority of which are stanols. Its phospholipid composition is complicated, but phosphatidylcholine and phosphatidylethanolamine are representatives [34]. The sterols, volatiles, and lipids in *Styela* sp. and *Phallusia* sp. were also determined, and he sterols mainly have a (22Z)-double bond. Relatively high concentrations of chlorinated compounds are the dominant volatiles, with *Styela* sp. rich in phenols and *Phallusia* sp. rich in hydrocarbons [13].

The lipid characteristics of *Pyrosoma atlanticum* were investigated, and structural polar lipids were found to be dominant. Sterols were the major neutral lipids, in addition to low contents of acylglycerols and free fatty acids. Among phospholipids, phosphatidylcholine (PC) was the most abundant, followed by phosphatidylethanolamine (PE), diphosphoglycerate (DPG), and others such as phosphatidylserine (PS), phosphatidylinositol (PI), also-PC (LPC), and sphingolipids. Saturated acids (16:0, 14:0) were the main fatty acids in DAG and TAG, while DHA and other MUFAs showed lower concentrations. In sterols, *24-methylcholesta-5,22E-dien-3β-ol* accounted for more than 22%, followed by cholesterol (*cholest-5-en-3β-ol*) at 12% and *24-methylcholesta-5,24(28)E-dien-3β-ol* at 11% [35].

In addition to protein and lipids, some tunicate species, such as *Ciona intestinalis*, have a unique capacity to accumulate metal ions from the environment, containing many different amounts of trace minerals, especially Zn, Mg, and V, which are also crucial to their exploitation [36]. It is well known that tunicates accumulate vanadium (V) from seawater via vanadocytes. Tatsuya et al. reported that in tunicates, vanadium tunicates is typically in the V_V_ state, but most vanadium in tunicates is reduced to V_III_ via V_IV_ during assimilation [37]. The quantification of the vanadium content in the tissues of numerous tunicates was ascertained by utilizing atomic absorption spectrometry, neutron activation analysis, and electron paramagnetic resonance (EPR). According to Michibata et al., tunicates of the suborder Phlebobranchia contained more V than those of the suborder Stolidobranchia. More particularly, the blood cells of the tunicate *Ascidia gemmata* contain the highest amounts of vanadium, reaching 350 mM, which is 107 times the concentration of seawater. *Ascidia gemmata* is thought to have the highest accumulation capacity of a specific metal in all living organisms [38]. Furthermore, *Halocynthia aurantium* can accumulate both essential elements necessary for physiological functions and metals with toxic properties. This characteristic of bioaccumulating trace elements makes *Halocynthia aurantium* a potential candidate for utilization as a bioindicator organism. Kosyanenko et al. investigated the levels of Fe, Zn, Mn, Cu, Pb, Ni, and Cd in various tissues of *Halocynthia aurantium*, including the tunic, stomach, gonads, muscular sac, and digestive gland. The highest concentrations of these elements were observed in the tunic (Mn), gonads (Cu), stomach (Zn and Cu), and digestive gland (Zn). Additionally, the Mn contents in the tunics of *Halocynthia aurantium* were higher than in other organs [39]. Consequently, the distribution of biologically significant Fe, Mn, Zn, and Cu was predominantly observed among the organs and tissues of *Halocynthia aurantium.* Based on the available studies mentioned above, it can be concluded that tunicate is generally a nutrient-rich marine biomass for the exploitation of food/feed applications. However, the presence of certain toxic elements should be considered to guarantee consumption safety.

## 5. Tunicates as a Source of Nutrients for Food and Feed Applications

Based on the literature review, tunicates can be a valuable marine biomass with a very high nutritional value which can potentially be exploited as human food and animal feed. Some tunicate species are edible and regarded as delicious seafood in some countries, such as South Korea, Brazil, and Japan. The most common edible species include *Halocynthia roretzi* [40], *Styela plicata* [41], *Styela clava* [42], and *Pyura michaelseni* [26] and some Mediterranean species, such as *Microcosmus vulgaris*, *Microcosmus sabatieri*, *Microcosmus hartmeyeri* and *Microcosmus polymorphus* [43].

### 5.1. Tunicate Species for Food Applications

**Figure 2:**
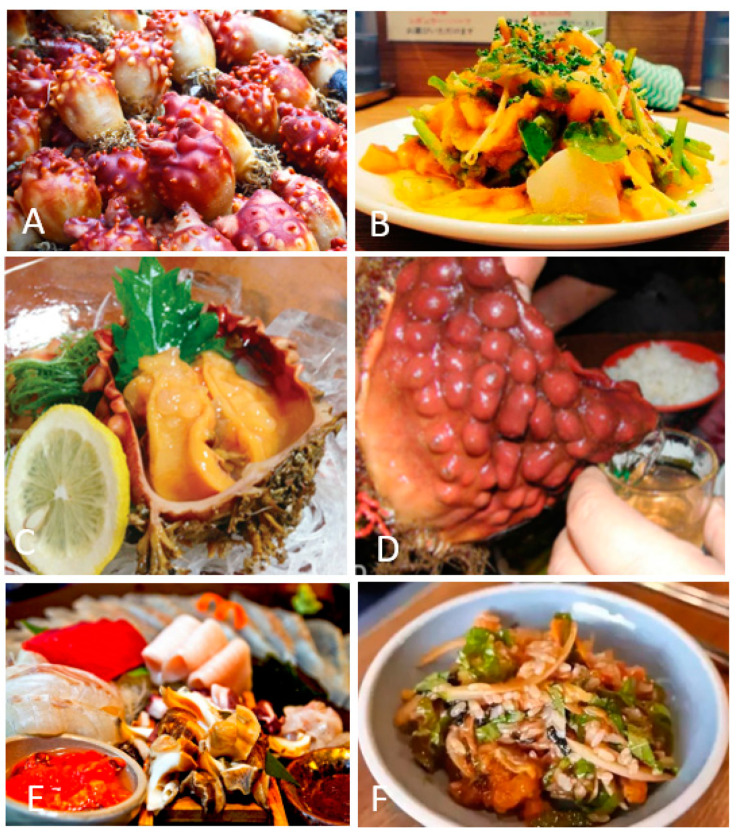
Tunicate species for food applications. (**A**) Fresh *Styela clava* as seafood [3]; (**B**) a dish of cooked *Styela clava*; (**C**) ready-to-eat *Microcosmus sabatieri*; (**D**) whiskey with *Halocynthia roretzi* flavor; (**E**,**F**) *Halocynthia aurantium* mixed with other seafood, such as whale meat, seal meat, and seaweed.

Tunicates are commonly consumed in Asia, Chile, and some Mediterranean countries, and the edible ones are preliminarily *Halocynthia roretzi*, *Halocynthia aurantium*, and *Microcosmus hartmeyeri*. These edible tunicate species are commonly available as fresh or dried products at seafood markets. As shown in Figure 2, edible tunicate species can be eaten in various forms. *Pyura chilensis*, locally called piure in Chile, is consumed domestically and exported to other countries, such as Sweden and Japan. *Halocynthia aurantium* is usually called the “sea peach” or “ice floe tunicate” (akaboya in Japan) and is dominantly farmed in Japan. Another edible tunicate species, *Halocynthia roretzi*, commonly called the “sea pineapple”, is widely farmed in Korea. In Korea, *Styela clava* is a delicious food; some people even consider it a functional food with an aphrodisiac effect. *Styela plicata* is consumed fresh in both Korea and some Mediterranean countries. *Microcosmus hartmeyeri* (harutoboya in Japan) is eaten in Japan. *Microcosmus sabatieri* and *Microcosmus vulgaris* are consumed as famous recipes in France, Italy, and Greece. Historically, the Māori in New Zealand consumed *Pyura pachydermatina*, which used to be a food source for aboriginal people living around Botany Bay, Australia.

### 5.2. Tunicate Species for Feed Applications

The search for alternative protein sources for fishmeal and plant-based ingredients is crucial for ensuring the sustainable development of the aquaculture industry. Tunicates are abundant and sustainably exploited marine animal organisms that have been exploited as a sustainable source of good-quality protein to substitute fishmeal and to serve as a nutrient component and attractant for fish, including Atlantic salmon [7]. In a recent study, the protein concentration in tunicates varied significantly among individuals and species within concentrations ranging between 2.09% and 14.09% of dry weight. This high variability in protein concentration from 2.4% to 10.7% of dry weight has also been present in different tunicate species in the Southern Ocean. In addition, they have a low lipid content, ranging from 0.5% to 3.8% [24,25]. Previous research suggested that tunicates can serve as a valuable food source for spiny lobster phyllosoma due to their relatively high tissue density, lack of complex body parts, and inability to escape predation [44]. It is crucial to study their processing and nutritional profiles when converting new biomass into protein components, both of which decide their level of use in aquatic feed. Samuelsen and his team conducted extensive research on the application of tunicate powder in aquatic feed, in which tunicate powder can replace 64% of fish meal in a formula without affecting the physical quality of the feed [44]. Meanwhile, the particle expansion rate and oil absorption ability of the feed are improved by the viscosity characteristics of the tunicate powder. A recent research study showed the nutritional limitations of tunicate meal (*Ciona intestinalis*) in feed for Atlantic salmon [45]. The results indicated that substituting half of the dietary fish meal protein with a tunicate meal at a concentration of 17% produced promising outcomes in terms of improved growth, tissue morphology, lipid and fatty acid profiles, and biometrics. The tunicate meal also reduced contaminants, including toxins (e.g., toxaphene) and heavy metals, in the whole bodies of salmon (e.g., arsenic and vanadium). Tunicate meals have low contents of protein (41%) and lipids (3%) but high concentrations of arachidonic acid, DHA, and EPA (3.5%, 17.2%, and 22.8% of total fatty acids, respectively), and they also have abundant essential amino acids. Compared to a control, a tunicate meal diet possessed a 3% lower protein apparent digestibility coefficient and a 0.7% lower fat apparent digestibility coefficient [46], this may be due to its high ash concentration in the test material, which was approximately 25%, including salt.

## 6. Tunicates as Sources of Bioactive Compounds

### 6.1. Natural Products from Tunicates

More than 1200 active molecules were identified from tunicates and tunicate-associated microbial species (Table 4) [47], confirming that they are abundant in various biologically active compounds [48]. For example, toxins [49], sphiningomyelins [50], and antimicrobial tunichromes [51] have been successfully extracted from different tunicate species. Lopez-Legentil et al. described two specific chemotypes in tunicate species. The pyridoacridines shermilamine B and kuanoniamine D were detected in the tunic, while their deacetylated forms were observed in the zooids. C9-unsubstituted pyrridoacridine ascidide was isolated from the purple morph of *Cystodytes dellechiajei* [52].

Wipf and Uto [53] separated the compound trunkamide A from *Lissoclinum* sp. and completely synthesized it [53]. Cyclic peptide bistratamides F–I were isolated from *Lissoclinum bistratum*, and their chemical structures were further validated using complete synthesis [54,55]. The pyridoacridine alkaloids arnoamines A and B were purified from the brownish purple tunicates of *Cystodytes* sp., and the total synthesis of these compounds was also realized [56]. According to Trieu et al., Eudistomins Y1–Y7, a subclass of frequently occurring and physiologically active β-carboline alkaloids, were wholly synthesized. Many of these alkaloids have been purified from *Eudistoma* sp. tunicates [57].

The screening of bioactive chemicals from tunicates is mainly used in anticancer and anti-inflammatory medications, with antimalarial medications and other medications ranking second and third [58]. Some peptides and alkaloids purified from tunicates show anticancer activities [59], while sulfated glycosaminoglycan (GAG) can be used for anticoagulation applications [60]. Plasmalogen has the potential to treat Alzheimer’s disease [61]. Therefore, tunicates are good sources of various bioactive components which are expected to provide health benefits for human beings [62]. Several molecules or derivatives have been used as clinical or marketed drugs, such as the marketed antitumor drugs trabetidine (ET-743/yondelis, used in soft tissue sarcomas and ovarian tumors), plitidepsin (used in multiple myeloma), lurbinectedin (used in small cell lung cancer) and midostaurin (used in acute myeloid leukemia), as well as the clinical drugs didemnin B, staurosporine, lestaurtinib, edotecarin, and becatecarin [63].

Some antibacterial, antifungal, hemolytic, and cytotoxic compounds were obtained by extracting *Lissoclinum fragile* with methanol. Pathogenic bacteria, including *Staphylococcus aureus*, *Vibrio parahaemolyticus*, *Vibrio cholerae*, and *Bacillus subtilis*, can be inhibited by a metabolite purified from *Eusynstyela tincta* [64]. A salt-tolerant peptide with antibacterial and antifungal activities was successfully extracted from hemocytes of *Ciona intestinalis*. Moreover, vanadyl sulfate and vanadium chloride with antibacterial properties were also obtained from this tunicate species [65].

**Table 4:** Bioactive substances from diverse tunicate-associated microbial species.

Tunicate Species	Chemical Compound	Function	References
*Polycarpaaurata*	Chrodrimanins A and H	Inhibited the activity of protein tyrosine phosphatase 1B	[66,67]
*Microbulbifer* sp.	Bulbiferates A and B	Antibacterial	[68]
*Unidentified ascidian* *from Tweed Heads*	Diterpene glycoside sordarin	Antifungal activity	[69]
*Ecteinascidia* *turbinata*	Ecteinamycin	Against microbial Clostridium difficile NAP1/B1/027	[70]
*Penicillium verruculosum*	Verruculides A and chrodrimanins A and H	Protein tyrosine phosphatase 1B inhibition	[71]
*Ecteinascidia* *turbinata*	Halomadurones C and D	Activated nuclear factor E2-related factor antioxidant response element (Nrf2-ARE)	[72]
*Pseudoalteromonas rubra*	Isatin	Antibacterial	[73]
*Pseudoalteromonas tunicata*	Tambjamine	Antifungal	[74]
*Ecteinascidia* *turbinata*	Bisanthraquinones 1 and 2	Cytotoxic against HCT116 cells Antimicrobial activity	[75]
*Synoicum prunum*	Prunolides A, B, and C	Inhibited the growth of HeLa cells	[40]
*Saccharopolyspora* sp.	JBIR-66	Cytotoxic	[76]
*Molgula manhattensis*	Granaticin, granatomycin D, and dihydrogranaticin B	Antimicrobial	[77]
*Clavelina picta*	Piclavines A–C	Antimicrobial	[78]
*Didemnum molle*	trichodermamides A and B	Antimicrobial	[79]
*Unidentified ascidian* *from Hiroshima*	Gifhornenolone A	Inhibited the activity of an androgen receptor	[80]
*Ectcinascidia turbinata*	Oxepinamide A	Anti-inflammatory activity on mouse ear edema	[81]

Except for the abovementioned tunicate species, Table 4 lists some bioactive compounds originating from different tunicate-associated microorganisms [5]. The Food and Drug Administration has authorized the use of didemnin B and Ecteinascidin 743 in the treatment of cancer, both of which were isolated from the tunicates’ symbiotic bacteria *Candidatus Endoecteinascidia frumentensis* [82]. As confirmed by several studies, didemnin B inhibits the growth of cancer cells by functioning as a cell cycle arrest agent. Cells in the G1/S phase respond to didemnin B more readily. It is well recognized that inhibitors of kinases, phosphatases, and elongation factors interfere with the transmission of mitogenic signals, which are the cause of the antiproliferative effectiveness of didemnin B [83].

### 6.2. Antimicrobial Activity

Antimicrobial medicines (such as antibiotics and antiviral), antifungal, and antiprotozoal medications are unquestionably essential to treating infectious disorders [84]. Many compounds extracted from various tunicate species show excellent antimicrobial activities, as listed in Table 5. In the hemocytes of *Styela clava*, clavanins A, B, C, and D peptides were extracted, all of which displayed excellent antimicrobial properties against both *Listeria monocytogenes* and *Candida albicans* [85]. The alkaloid tambjamine, extracted from *Pseudoalteromonas tunicate,* is a yellow antifungal pigment [86]. With high levels of antibacterial action against *Staphylococcus aureus* and *Pseudomonas aeruginosa*, halocidin can be extracted from *Halocynthia aurantium*. In addition, halocyntin and papillosin, as antibacterial peptides which were purified from *Halocynthia papillosa*, eliminated both Gram-positive and harmful bacteria [87].

New metabolites based on β-carbolines, namely, didemnolines (A–D), were isolated from the tunicates *Didemnum* sp. These tunicates were predominantly found in the Northern Mariana Islands, United States [99]. Didemnoline C exhibits zones of growth inhibition of 9 mm and 7 mm growth inhibition against *Staphylococcus aureus* and *E. coli*, respectively. Didemnolines A also exhibits potent cytotoxicity against human epidermoid carcinoma (KB cells) at a 0.28 lg/mL concentration. The colonial tunicates *Eudistoma* sp., which grow on mangrove roots in Chuuk, Micronesia, were utilized to extract the eudistomins W and X [100].

Arenimycin is a benzo[α] naphtacene quinone molecule with considerable antibiotic activity produced by the actinobacterium *Salinospora arenicola*, which is associated with the tunicates *Ecteinascidia turbinata*. With an Minimum Inhibitory Concentration (MIC) of less than 1 g/mL, arenimycin demonstrated the potent inhibition of bacterial growth in multidrug-resistant Staphylococcus strains, *Enterococcus faecalis*, and *Enterococcus faecium*. Arenimycin also showed an MIC of 1 g/mL against *M. bacillus*. They were also effective against a human adenocarcinoma cell line (HCT-116), with an IC_50_ value of 1.16 g/mL and cytotoxic effects, although a nonselective mechanism of action has been proposed [101].

**Figure 3:**
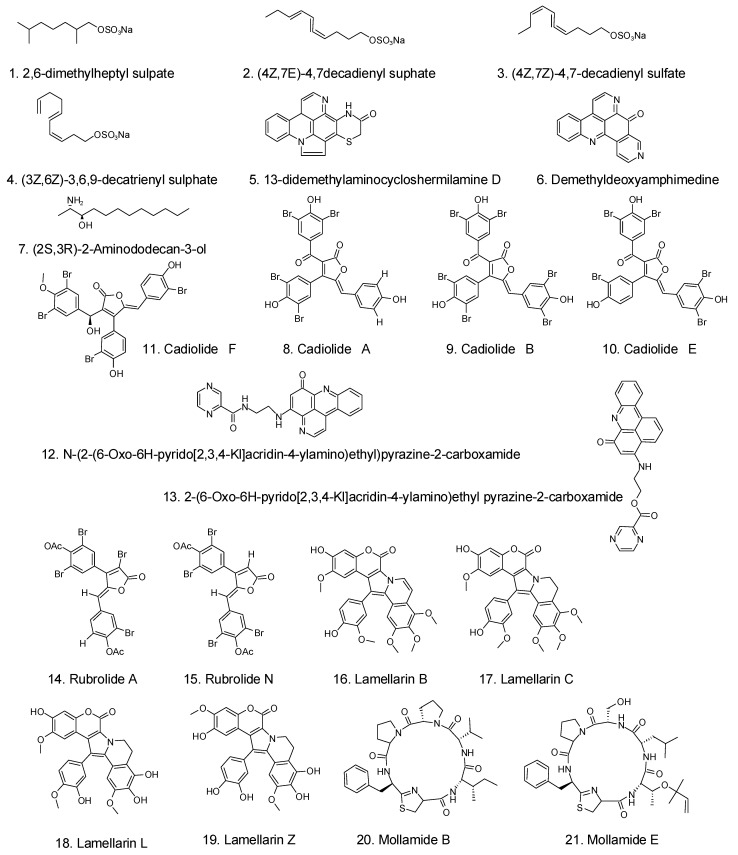
Compounds extracted from tunicates with antimicrobial potential [9].

Ascididemin-related pyrido [2,3,4-kl] acridin-6-one pyridoacridine alkaloids were discovered in the tunicates *Lissoclinum notti*, which were found on the coast of New Zealand [102]. Among all the identified metabolites, 2-(6oxo-6H-pyrido[2,3,4-kl]acridin-4-ylamino)ethyl pyrazine2-carboxylate ((12), Figure 3) and N-(2-(6-oxo-6H-pyrido[2,3,4-kl] acridin4-ylamino)ethyl)pyrazine-2-carboxamide ((13), Figure 3) inhibited the growth MIC of *Mycobacterium tuberculosis* H37Rv at 2 μM and exhibited cytotoxicity against Vero and P388 cells at >25 μM.

### 6.3. Antitumor and Anticancer Activities

**Figure 4:**
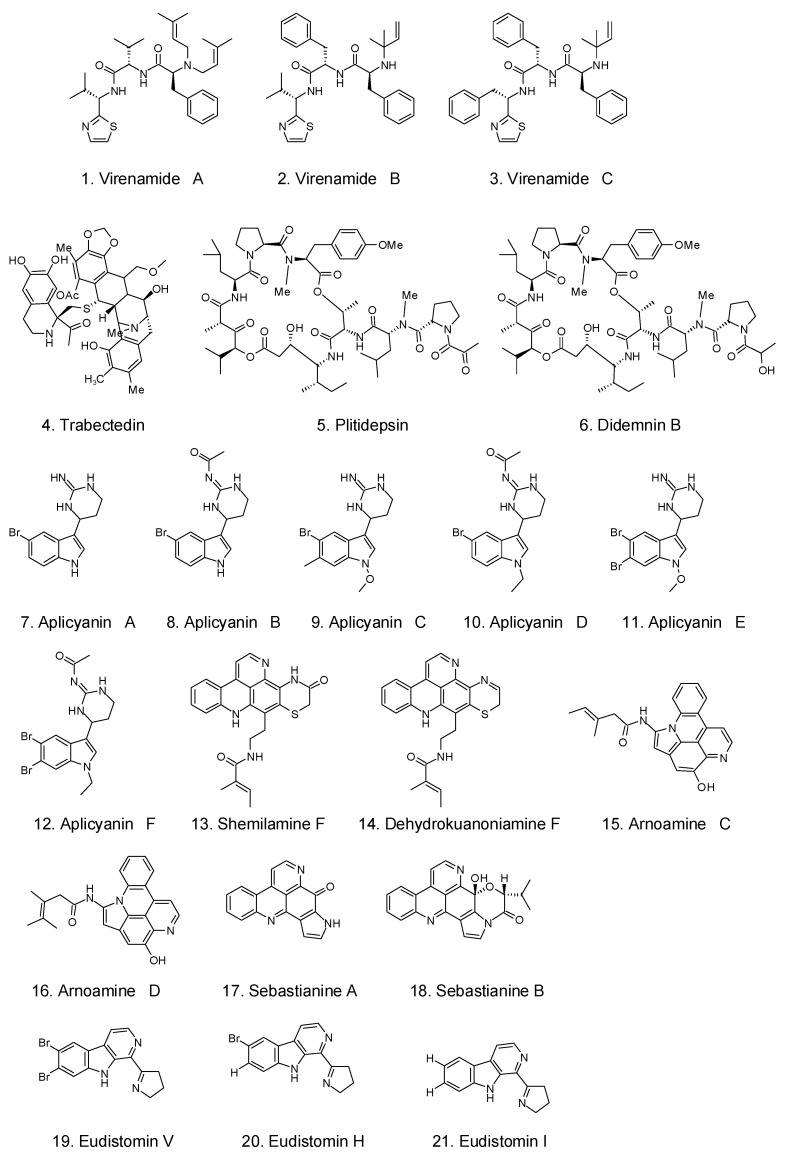
Some crucial anticancer medications from tunicates and the related microorganisms [5].

In addition to the well-demonstrated antimicrobial activities, some compounds extracted from tunicates provide great anticancer properties. Figure 4 shows some crucial anticancer medications derived from tunicates and the related microorganisms. Zhu et al. [103] reported that aqueous extracts from the edible portions of *Halocynthia roretzi* dramatically reduced the viability of HepG-2 cells. By using semipreparative HPLC to further separate the extracts, the active components were a mixture of fatty amides comprising hexadecanamide, stearamide, and erucamide, as determined via ultra-high-performance liquid chromatography (UHPLC)-MS/MS [103].

Trabectedin, ET-743, an alkaloid discovered in the orange tunicate *Ecteinascidia turbinate*, was the first anticancer drug approved in 2015 by the FDA for the treatment of soft tissue sarcoma in the United States [47]. Trabectedin originated from an *E. turbinata* symbiotic bacteria, *Candidatus Endoecteinascidia frumentensis*, and shows excellent activity in killing cancer cells in soft tissue, breast, and ovarian organs. The DNA minor groove is bound by trabectedin, which also causes DNA cleavage and alters the tumor microenvironment. Trabectedin prevents tumor cells from neoangiogenesis and metastasis, which are crucial in the treatment of cancer [104].

A depsipeptide, plitidepsin, extracted from *Aplidium albicans*, has been tested in phase II clinical trials, showing great anticancer activity against various cancers located in the kidney and breast [105]. Many alkaloid ecteinascidins were extracted from *Ecteinascidia turbinata*, demonstrating anticancer activity due to their capacity to form covalent bonds with DNA molecules [106]. Another drug in phase II trials, Didemnin B, shows good anticancer activity against leukemia P388 cells [107]. After hydrolysis of *Styela clava* by alcalase, a hydrolysate with good antitumor properties was obtained, with high ABTS and 1,1-diphenyl-2-picrylhydrazyl (DPPH) radical scavenging activities [59]. *Clavelina picta* is a source of Clavepictine A and B, which display anticancer properties against human solid tumor cell lines [108]. Lamellarin sulfates and dihydrochloride purified from *Didemnum ternerratum* and *Polycarpa clavata*, respectively, can inhibit colon cancer cells. Diplamine, an orange alkaloid, was purified from *Diplosoma* sp. and showed anticancer activity against leukemia cells. Two staurosporine derivatives (2-hydroxy-7-oxostaurosporine and *3-hydroxy-7-oxostaurosporine*) were extracted from *Eudistoma vannamei* via moderately polar and methanol solutions, respectively. When these two compounds were mixed, the mixture exhibited excellent anticancer properties against many tumor cells [109]. From *Cynthia savignyi*, an effective antitumor component, cynthichlorine, was extracted as an alkaloid, whose LD50 was 48.5 µg/mtetracycliceracyclic alkaloids. The cystodytins A, B, and C were found in the Okinawa tunicate *Cystodytes dellechhadi* and have anticancer properties. In addition, other extractives from tunicates, such as the peptides halocyamine A and B, dehydrodidemnin B, topoisomerase II-inhibiting ascididemin, and the derivatives of siladenoserinols A and B, also demonstrated antitumor activity [110]. The antitumor activity of derivatives of siladenoserinols A and B, extracted from didemnid tunicates, was proved by inhibiting the p53–Hdm2 interaction. In 2014, researchers from India isolated a substance that inhibits cells from the extract of the sea squirt *Eudistoma viride*. After identification, it was found to be an indole alkaloid bromide that can induce apoptosis in HeLa cells.

### 6.4. Antidiabetic Activity

The long-term metabolic disorder diabetes is a rapidly spreading epidemic with serious social, health, and economic ramifications. According to the prediction of the World Health Organization, more than 439 million people will have diabetes-related health issues in 2030 [111]. *Halocynthia roretzi* contains unsaturated fatty acids such as DHA and EPA [112]. The addition of Halocynthia roretzi lipid extracts also exerted hypolipidemic and hypoglycemic effects in diabetic mice [113]. Herdmanines E-L and (-)-(R)-leptoclinidamine B are two novel congeners of antidiabetic amino acid derivatives that have been identified. In a cell-based luciferase reporter experiment, indoleglyoxylyl derivatives of herdmanine K demonstrated potent PPAR-c activation in rat liver cells (Ac2F) at concentrations of 1 and 10 g/mL [114].

One of the most critical factors in managing diabetes is the delay in starch digestion caused by the suppression of enzymes such as α-amylase. Pancreatic α-amylase inhibitors prohibit the breakdown of carbohydrates, thus slowing down the absorption of glucose and lowering postprandial blood glucose levels. Prabhu et al. used an in vitro model to detect the inhibition of α-amylase in ethyl acetate, methanol extracts, and acetone extracts of 10 kinds of tunicates, *Phallusia mammillata*, *Phallusia arabica*, *Microcosmus squamiger*, *Polyclinum* sp., *Ascidia ahodori*, *Microcosmus* sp., *Trididemnum savignii*, *Polyclinum aurantium*, *Ascidia* sp., and *Didemnum vexillum*, among which *Phallusia mammillata* showed the highest levels of amylase inhibition activity without causing any adverse side effects, achieving the highest degree of inhibition (68%) at a concentration of 300 g/mL [115].

**Figure 5:**
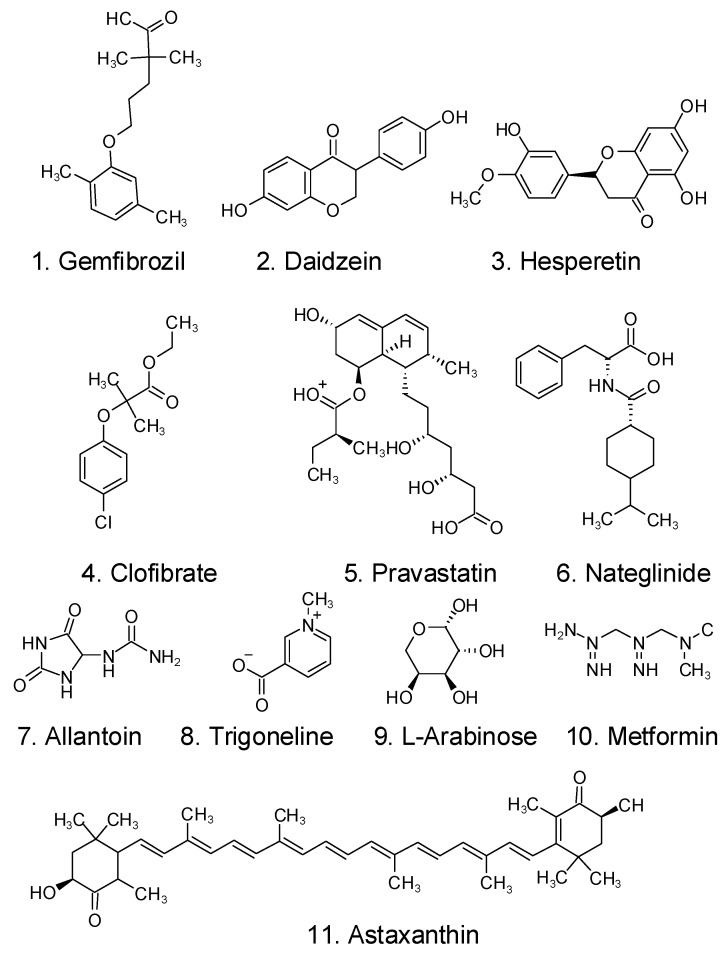
Structural map of 11 antidiabetic molecules in the metabolome of *Halocynthia roretzi* tissue [116].

Zhu et al. examined the metabolome of *Halocynthia roretzi* tissue by using UHPLC–MS-MS, discovering 11 active compounds that contribute to the metabolism of lipids and blood glucose, as shown in Figure 5 [116]. Some molecules share structural similarities with pharmaceuticals now on the market, including metformin and gemfibrozil (two well-known medications used to treat type 2 diabetes mellitus and dyslipidemia, respectively) [116].

### 6.5. Antioxidant Activity

**Figure 6:**
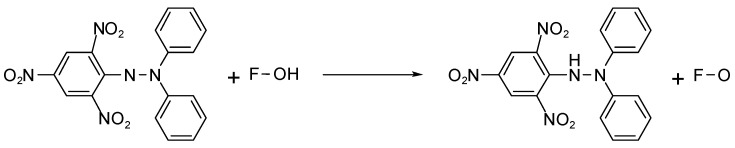
The antioxidants capture 1,1-diphenyl-2-picrylhydrazyl radicals via proton donation [117].

Antioxidants are substances that can prevent the oxidation of other molecules. Free radicals obtain one or more electrons from these antioxidant chemicals which prevents the damage that they produce [117]. The antioxidant activity of the lamellarin (lamellarin and I) alkaloids isolated from the Indian tunicate *Didemnum obscurum* was tested using the DPPH technique, demonstrating a very strong antioxidant effect [118]. Proton contributions to radicals are the mechanism through which antioxidants capture DPPH radicals, as depicted in Figure 6 [117]. Elya et al. employed the DPPH approach to identify the antioxidant activity of tunicates. N-hexane, ethyl acetate, and water were used to extract the dibutyl ester. Accelerated column chromatography further separated the fractions with the highest levels of antioxidant activity. The *Didemnum* sp. tunicates’ methanol extract showed the best antioxidant activity. The *Didemnum* sp. extract was fractionated, and the results revealed that the ethyl acetate fraction exhibited the maximum antioxidant activity. The rapid column chromatography fractionation of the ethyl acetate fraction revealed that fraction VI had the best antioxidant activity. Alkaloids, steroids/triterpenoids, saponins, and glycosides were present in the most active parts [117].

Ma et al. extracted antioxidant peptides from the inner body tissues of *Halocynthia roretzi* using enzymatic hydrolysis and ultrafiltration [119]. A 6-OHDA-induced oxidative injury PC12 cellular model was established to assess the neuroprotective effects of these novel antioxidant peptides. These findings revealed that six new antioxidant peptides were derived from *Halocynthia roretzi*, including Phe-Gly-Phe (FGF), Trp-Leu-Pro (WLP), LeuPhe-VAL (LFV), Val-Phe-Leu (VFL), Leu-Gly-Phe (LGF), and Ile-Ser-Trp (ISW). WLP scavenged excess ROS and increased antioxidant enzyme activity through oxidative stress pathways, and preincubating cells with the antioxidant peptide WLP showed a considerable cytoprotective ability against 6-OHDA-induced oxidative stress in PC12 cells [119].

### 6.6. Anti-Inflammatory Activity

Alkaloids derived from tunicates represent a significant class of compounds with anti-inflammatory effects [120]. Inflammation is an intricate physiological response. Vascular alterations, such as vasodilation, increased permeability, and reduced blood flow are caused by a range of signaling molecules generated by white blood cells, macrophages, monocytes, and mast cells, as well as damaging stimuli such as complement factors that activate pathogens to generate either harmful chemicals or cell damage. Dermatan sulphate from the Brazilian coast’s *Styela plicata* has anti-inflammatory effects [121]. At various dosages up to 200 mg/kg, the crude methanol molecule isolated from *Eudistoma viride* showed modest anti-inflammatory efficacy [122].

When 5-HT formalin, mustard, bradykinin, carrageenan, egg white, or histamine was injected into the dorsum of a rat’s foot, an immediate inflammatory reaction was triggered in the form of paw edema in mice. The anti-inflammatory effects of the methanol extracts of two tunicates, *Eudistoma ovatum* and *Didemnum perlucidum*, were evaluated using a carrageenan-induced edema foot model. Their influences on hematological parameters (red blood cells, white blood cells, and platelets), tumor necrosis factor (TNF), interleukin-6 (IL-6), and antioxidant enzymes (SOD, GSH, LPO) were investigated. In their study, the researchers discovered that *Eudistoma ovatum* and *Didemnum perlucidum* contain compounds that exhibited anti-inflammatory activity in a rat model of carrageenan-induced paw edema. Furthermore, the methanolic extract of *Eudistoma ovatum* demonstrated a more potent anti-inflammatory effect than other extracts [58].

**Figure 7:**
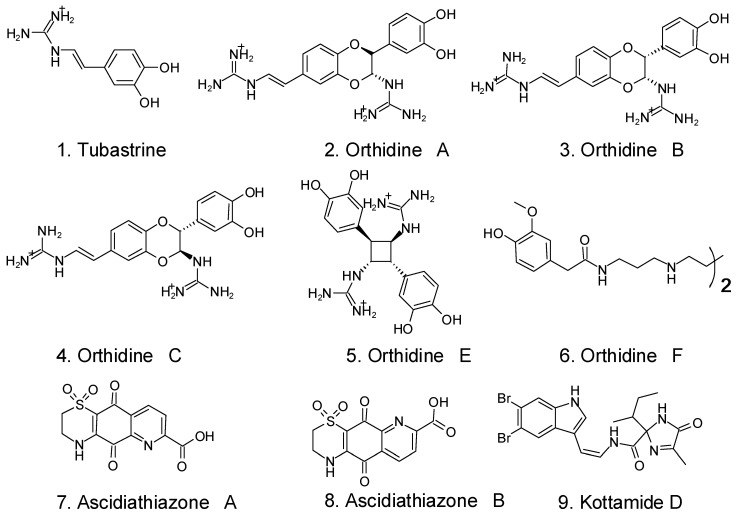
Some tunicates’ alkaloids with anti-inflammatory activity [120].

The tunicate *Aplidium orthium*’s alkaloids tubastrine (1) and the orthidines A (2), B (3), C (4), E (5), and F (6) were proven to have anti-superoxide activity against phorbol-12-myristate-13-acetate (PMA)-stimulated neutrophils in vitro as well as anti-superoxide activity against a gouty arthritis model in vivo [123]. Additionally, tubastrine and orthidine F inhibited neutrophil invasion in this in vivo model. Both in vitro and in vivo murine gout models using ascidiathiazones A (7) and B (8), two thiazone-containing quinolinequinone alkaloids derived from *Aplidium* spp., reduced the formation of superoxide by PMA-stimulated neutrophils [124]. In vitro, PMA and N-formylmethionyl-leucyl-phenylalanine (fMLP)-activated neutrophils were able to suppress superoxide generation via the imidaloze-containing alkaloid kottamide D (9) from the tunicate *Pycnoclavella kottae* [125]. The chemical formulas of the alkaloids are shown in Figure 7.

Herdmanines A-D, biologically active derivatives of amino acids, were discovered in the tunicate *Herdmania momus*. Notably, herdmanines A–C contain the uncommon D-form of arginine [126]. These compounds, particularly herdmanines C and D, demonstrate significant suppression of nitric oxide production, with IC_50_ values of 96 μM and 9 μM, respectively. Furthermore, these compounds can potentially inhibit the mRNA expression of inducible nitric oxide synthase (iNOS). Herdmanine D exhibits a potent inhibition of the mRNA expression of the proinflammatory cytokine IL-6 [9].

### 6.7. Bioactive Compounds Explored in Clinical Trials

The natural products chemistry research field has experienced a notable shift toward practical applications, specifically focusing on biologically active compounds with pharmacological value. As of 2022, only a limited number of drugs derived from tunicates were sanctioned by regulatory authorities, such as the natural drug trabectedin (ET-743/Yondelis^©^) and two synthetic drugs, the trabectedin derivative lurbinectedin (PM01183/Zepsyre^©^) and the staurosporine synthetic derivative midostaurine (PKC-412/CGP41251/N-benzoylstaurosporine/Rydapt^©^) (Table 6) [127].

## 7. Conclusions and Prospects

Despite their abundance in nature, the exploration of tunicates as a new type of marine bioresource is still limited. In recent years, the technology used to cultivate tunicates has matured, and tunicates have gradually become highly nutritious seafood that everyone can eat. Currently, many species of edible tunicates are cultured, such as *Halocynthia roretzi*, *Halocynthia aurantium*, and *Microcosmus hartmeyeri*. *Halocynthia roretzi* is cultured in Korea using longline aquaculture techniques. Wawrzyniak et al. created a loop aquaculture system to serve as a model for extending tunicate culture. Therefore, the consumption of tunicates as seafood or at least as animal feed will be expanded. To exploit tunicates as valuable marine biomass economically and practically, a detailed study of the chemical compositions of tunicates is critical. On one hand, the chemical compositions of different tunicate species should be determined to identify their nutritional value and promote their further development. On the other hand, detailed chemical characterizations of the different fractions in tunicates must be further analyzed with the expectation of better understanding the location of various nutritional compounds and facilitating their future uses in the food and feed fields.

Bioactive compounds extracted from tunicates encompass novel metabolites, including alkaloid derivatives, peptides, polyketides, quinones, and steroids. These compounds exhibit a wide range of bioactivities, spanning antibacterial, antidiabetic, anti-inflammatory, antioxidant, antitumor, and anticancer properties, both in vitro and in vivo [9]. Tunicates represent a rich source of new drugs. However, contemporary research predominantly focuses on a few selected species, such as *Aplidium* sp., *Synoicum* sp., and *Eudistoma* sp. [9]. To further expand their application, it is necessary to conduct in-depth research involving the bioactive compound analysis of other tunicates. With advancements in science and technology, the contemporary utilization of sophisticated sampling techniques, advanced analytical tools, novel genetic methods, molecular biology tools, metabolomics methods, natural library databases, computational biology, biosynthesis technology, and other emerging technologies, the investigation into tunicates as a source of pharmaceutical candidates will be expedited. In addition, most of the active ingredients found in tunicates until now are mainly in the laboratory research stage, and their actions are still unclear. Therefore, further research is needed to evaluate the safety and effectiveness of these compounds as potential drug candidates.

## Figures and Tables

**Table 2:** General chemical compositions of the outer shells and internal organs of tunicate species.

Tunicates Species	*Ciona intestinalis*	*Ascidia* sp.	*Ciona intestinalis*	*Halocynthia roretzi*	*Salpa thompsoni*	*Styela clava*	*Pyura michaelseni*
Crude protein (%)	30.86	41.34	51.82	43.05	4.40	8.30	12.30
Crude lipid (%)	-	-	-	-	-	2.10	1.40
Cellulose (%)	57.67	37.57	37.29	52.59	-	-	-
Lipids calculated by fatty acid (%)	0.35	0.98	0.42	0.18	5.70	-	-
Total sugar (%)	-	-	-	-	-	1.50	2.70

**Table 3:** Fatty acid compositions of certain tunicate species.

Fatty Acid (%)	*Styela plicata*	*Ascidia* sp.	*Ciona intestinalis*	*Halocynthia roretzi*	*Halocynthia aurantium*	*Cystodytes violatinctus*	*Pyrosoma atlanticum*	*Cystoseira tamariscifolia*	*Cynthia squamulata*
C12:0	-	-	-	-	-	1.20	-	2.55	2.36
C14:0	0.68	3.76	2.08	4.10	4.90	4.50	6.93	3.40	3.71
C15:0	2.04	1.18	0.83	6.10	4.60	1.50	1.40	4.37	4.69
C16:1	4.08	6.35	8.33	2.10	-	1.26	2.51	5.36	6.14
C16:0	15.31	20.94	11.67	25.40	18.10	21.20	16.48	5.08	5.83
C17:0	7.48	1.18	1.25	7.20	6.50	4.80	0.77	6.22	6.47
C18:4	0.34	1.41	0.83	-	-	-	3.95	11.71	12.14
C18:3	-	-	-	-	-	-	0.11	9.84	9.76
C18:2	1.36	0.71	1.67	-	-	-	2.34	9.09	8.97
C18:1	15.65	25.41	14.58	-	-	20.0	6.78	8.25	8.85
C18:0	11.22	12.71	6.25	16.80	18.60	24.80	1.19	7.75	8.53
C19:0	-	-	-	-	2.30	3.60	-	-	-
C20:5	25.85	6.82	24.58	-	-	-	0.19	21.41	20.74
C20:4	0.34	2.35	0.42	-	-	-	-	-	-
C20:2	1.7	1.65	0.83	-	-	-	0.13	-	-
C20:1	3.06	7.76	10.83	-	-	-	0.75	-	-
C20:0	0.34	0.47	1.67	2.50	5.00	3.10	0.19	-	17.35
C21:0	-	-	-	-	-	2.30	-	-	-
C22:6	5.78	2.59	12.92	-	-	-	-	-	40.13
C22:1	3.4	2.12	1.33	4.10	-	-	-	-	-
C22:0	1.36	2.59	0.33	-	25.80	0.40	0.06	-	-
C23:0	-	-	-	-	4.50	-	-	-	-
C24:0	-	-	-	-	2.10	-	-	-	-
SFA	38.43	42.83	24.08	-	-	-	-	-	-
MUFA	26.19	41.64	35.07	-	-	-	-	-	-
PUFA	35.37	15.53	41.25	-	-	-	-	-	-
UFA	61.56	57.17	76.32	-	-	-	-	-	-
ω-3 ratio	31.97	10.82	38.33	-	-	-	-	-	-
ω-6 ratio	3.4	4.71	2.92	-	-	-	-	-	-
ω-6/ω-3 ratio	0.11	0.44	0.08	-	-	-	-	-	-

**Table 5:** Antimicrobial compounds extracted from tunicates.

Tunicate Species	Bioactive Compound	Activity against	Reference
*Cynthia savignyi*	Cynthichlorine	*A. radiobacter*, *E. coli*, *P. aeruginosa*, *Botrytis cinerea*, *Verticillium albo atrum*	[88]
*Eusynstyela tincta*	Clavanins	*E. coli*, *L. monocytogenes*, *C. albicans*	[89]
*Pseudodistoma antinboja*	Cadiolides J–M	Gram-positive bacteria	[90]
*Styela clava*	Clavanins A–D	*Pathogenic L. monocytogenes*, *C. albicans*	[91]
*Diplosoma* sp.	Diplamine	*E. coli*, *S. aureus*	[92]
*Eusynstyela latericius*	Eusynstyelamides A and B	*S. aureus*	[64]
*Halocynthia papillosa*	Halocyntin and papillosin	Gram-positive and Gram-negative marine bacteria	[87]
*Halocynthia roretzi*	Halocyamines A and B	Bacteria and yeasts	[93]
*Halocynthia aureum*	Halocidin	Methicillin-resistant *Staphylococcus aureus* and multidrug-resistant *Pseudomonas aeruginosa*	[94]
*Eusynstyela tincta*	Kuanoniamine A	*B. subtilis*, *E. coli*, *S. aureus*, *V. cholerae*, and *V. parahaemolyticus* and fungi *A. fumigatus* and *C. albicans*	[89]
*Ciona intestinalis*	Salt-tolerant peptide	Gram-negative and Gram-positive bacteria	[95]
*unidentified tunicate*	Talaropeptides A and B	Gram-positive bacteria, *Bacillus subtilis*	[96]
*Didemnum* sp.	Terretriones C and D	*C. albicans*	[97]
*Pseudoalteromonas tunicata*	190-kDa protein	Marine isolates	[98]

**Table 6:** Some bioactive compounds of tunicates utilized in clinical trials.

Bioactive Compound	Tunicate Species	Natural Product or Derivative	Biosynthetic Class of Agent	Molecular Target	Disease Area	References
Trabectedin (ET-743/Ecteinascidine 743/Yondelis^©^)	*Ecteinascidia turbinate*	Natural product	NRPS-derived alkaloid	Minor groove of DNA	Cancer	[128]
Lurbinectedin (PM01183/Zepzelca^©^)	*Ecteinascidia turbinat*	Derivative; trabectedin analog	NRPS-alkaloid	Minor groove of DNA and nucleotide excision repair	Cancer	[129]
Midostaurine (PKC-412/CGP41251/Rydapt^©^)	Tunicates	Semisynthetic analogue of staurosporine	Indolocarbazole	Flt-3 and PKC	Cancer	[130]
Plitidepsin (dehydrodidemnin B/Aplidine^©^)	*Aplidium albicans*	Natural product	Cyclic depsipeptide	Rac1 and JNK activation	Cancer	[104,131]
Lestaurtinib (CEP-701)	Tunicates	Synthetic analogue of Staurosporine	Indolocarbazole	Flt-3, JAK-2, Trk-A, Trk-B, and Trk-C	Cancer	[132]
Enzastaurin (LY317615)	Ascidian	Synthetic analogue of Staurosporine	Indolocarbazole	PKCβ and GSK-3β	Cancer	NCT00332202
Becatecarin (NSC 655649)	Tunicates	Synthetic analogue of Staurosporine	Indolocarbazole	Potent stabilizers of DNA	Cancer	[133]
Didemnin B	*Trididemnum solidum*	Natural product	Cyclic depsipeptide	Anti-viral agent	Cancer	[134]

CTN: clinical trial number.

## Data Availability

Data are contained within the article.
